# Prognostic impact of standard laboratory values on outcome in patients with sudden sensorineural hearing loss

**DOI:** 10.1186/1472-6815-14-6

**Published:** 2014-07-09

**Authors:** Julia Wittig, Claus Wittekindt, Michael Kiehntopf, Orlando Guntinas-Lichius

**Affiliations:** 1 Department of Otorhinolaryngology, Jena University Hospital, Lessingstrasse 2, Jena D-07740, Germany; 2 Present address: Department of Otorhinolaryngology, University Giessen, Giessen, Germany; 3 Institute of Clinical Chemistry and Laboratory Diagnostics, Jena University Hospital, Jena, Germany

**Keywords:** Serology, Blood value, Outcome, Prognostic marker, Hearing loss

## Abstract

**Background:**

Aim of the present study was to evaluate prognostic factors, in particular standard laboratory parameters, for better outcome after idiopathic sudden sensorineural hearing loss (SSNHL).

**Methods:**

Using a retrospective review, 173 patients were included presenting between 2006 and 2009 with unilateral SSNHL, ≥30 dB bone conduction in three succeeding frequencies between 0.125 to 8 kHz in pure tone audiometry (PTA), and a time interval between first symptoms and diagnostics ≤ 4 weeks. Hearing gain of <10 dB versus ≥10 dB in the affected ear in 6PTA values was the primary outcome criterion. Univariate and multivariate statistical tests were used to analyze predictors for better outcome.

**Results:**

The initial hearing loss was 50.6 ± 27.2 dB. The absolute hearing gain was 15.6 ± 20.1 dB. Eighty-one patients (47%) had a final hearing gain of ≥10 dB. Low-frequency hearing loss (*p* <0.0001); start of inpatient treatment <4 days after onset (*p* = 0.018); first SSNHL (versus recurrent SSNHL, *p* = 0.001); initial hearing loss ≥ 60 dB (*p* < 0.0001); an initial quick value lower than the reference values (*p* = 0.040); and a pretherapeutic hyperfibrinogenemia (*p* = 0.007) were significantly correlated to better outcome (≥10 dB absolute hearing gain). Multivariate analysis revealed that first SSNHL (*p* = 0.004), start of treatment <4 days after onset (*p* = 0.015), initial hearing loss ≥ 60 dB (*p* = 0.001), and hyperfibrinogenemia (*p* = 0.032) were independent prognostic factors for better hearing recovery.

**Conclusion:**

Better hearing gain in patients with hyperfibrinogenemia might be explained by the rheological properties of the applied therapy and supports the hypothesis that SSNHL is caused in part by vascular factors.

## Background

Idiopathic sudden sensorineural hearing loss [SSNHL) is defined as unexplained unilateral sensorineural hearing loss of 30 dB HL or greater over 3 continuous frequencies with onset over a period of less than 72 hours and with no marked vestibular symptoms [1]. SSNHL has an estimated incidence between 160 and 400 per 100,000 persons per year, i.e. much higher than assumed in older studies [2,3]. The causes of SSNHL are speculative and probably multifactorial [4]. Cardiovascular disease, cigarette smoking, and hypertension appear to be the most common risk factors associated with SSNHL [2,4]. Advanced age, severe hearing loss, heredity, audiogram shape, and presence of vertigo seem to be significant negative prognostic factors [4,5]. Most studies analyzing prognostic factors do not evaluate if the therapy itself applied to the patients with SSNHL has an influence on these prognostic factors. Furthermore, most studies include a variety of therapy regimes and sometimes also patients who did not receive any therapy. This makes it difficult to interpret the concrete role of the discovered risk factors. In a recent study using a uniform standardized therapy consisting of carbogen inhalation and oral prednisone, the prognostic factors for better recovery were severity of initial hearing loss, presence of vertigo, time between onset and treatment, the hearing of the other ear, and the audiogram shape [5].

Although especially vascular factors are constantly discussed to be related to the etiology of SSNHL, it is surprising that so far the prognostic impact of the entire range of routine laboratory values has not been evaluated systematically. Therefore, the present study investigated whether patients with SSNHL and its comorbidity also influence routine pretherapeutic laboratory values and whether these values have prognostic influence on hearing recovery after a standardized combined glucocorticoid and rheological therapeutic regime.

## Methods

### Patients

A standardized retrospective analysis was performed in the Department of Otorhinolaryngology of the University Hospital Jena in Germany. The study protocol was approved by the institutional ethics committee of the Friedrich-Schiller University, Jena, Germany. All adult patients who were treated for unilateral idiopathic sudden sensorineural hearing loss between 2006 and 2009 were included in the database for this study. Prerequisite was a differential diagnostic evaluation excluding a specific etiology (like head trauma, vestibular schwannoma) explaining the sudden hearing loss. All patients received a brainstem electrical response audiometry (BERA). If the BERA was pathologic, a magnetic resonance imaging (MRI) of the head and cerebellopontine angle was performed. Further inclusion criterion was that at least 2 pure-tone audiograms were available: the first at presentation prior to initiation of therapy, and a second after therapy. If more than one follow-up audiogram was available, the last audiogram was taken for analysis. Follow-up audiometry was stopped when no further hearing improvement was seen. Exclusion criteria were: Hearing loss <30 dB bone conduction in three succeeding frequencies between 0.125 to 8 kHz as revealed by pure tone audiometry; time interval between first symptoms and diagnostics > 4 weeks; acute bilateral hearing loss; combination with acute vestibular hypofunction (excluded by caloric vestibular testing); history of chronic ear disease (like otosclerosis, chronic otitis media, Menière’s disease). A search of the patients’ electronic charts was performed, and the following variables were obtained: age, sex, smoking behavior (yes/no), Charlson comorbidity index [6], presentation of a metabolic syndrome (if patient had ≥3 of the following diseases: diabetes mellitus, hypertension, adiposity, atherosclerosis, gout, hyperlipidemia), presentation of cardiovascular risk factors (if patients had at least 1 of the following diseases/history of diseases: vein thrombosis, apoplexy, cardiac infarction, diabetes mellitus, hypertension, hyperlipidemia) and tinnitus (yes/no).

All patients were hospitalized. Treatment followed the German guideline for sudden idiopathic sensorineural hearing loss as 1-week combination of glucocorticoid and rheological therapy with pentoxifyllin [6,7]. Mainly, a tapered course of oral corticosteroids is regarded as standard treatment [1].

### Audiometric assessment

Audiometric evaluation included air conduction and bone conduction thresholds on the affected and the contralateral side revealed by pure tone audiometry. The pure tone average (PTA) was calculated from the results of bone conduction at 0.25, 0.5, 1, 2, 4, and 6 kHz (6PTA). The severity of the hearing loss was described exactly according to Cvorovic et al. [5] as 1) mild, PTA of 15 to 39 dB; 2) moderate, PTA of 40 to 59 dB; 3) severe, PTA of 60 to 79 dB; 4) profound, PTA of 80 to 100 dB; and 5) deaf, PTA of greater than 100 dB [5]. Furthermore, the pattern of the initial audiogram was categorized into 1 of 4 types [5]: Low frequencies were defined as 0.5 kHz or less, midfrequencies as greater than 0.5 and 2 kHz or greater, and high frequencies as greater than 2 and 8 kHz or less. The following types of audiograms were defined: 1) low frequency, ascending, greater than 15 dB HL from the poorer low-frequency thresholds to the higher frequencies; 2) midfrequency, U-shaped, greater than 15 dB HL difference between the poorest thresholds in the midfrequencies and those at higher and lower frequencies; 3) high frequency, descending, greater than 15 dB HL difference between the mean of 0.5 and 1 kHz and the mean of 4 and 8 kHz; 4) flat, less than 15 dB HL difference between the mean of 0.25-, 0.5-kHz thresholds, the mean of 1 and 2 kHz, and the mean of 4 and 8 kHz; and 5) total deafness, hearing loss of 100 dB or more in 0.5, 1, 2, and 4 kHz.

Hearing gain was expressed as absolute hearing gain (∆6PTA; dB values) from initial PTA minus dB values from final PTA. If a negative value was calculated, the hearing gain was set to zero. For calculation of the relative hearing gain, the absolute gain ∆PTA was divided by the initial PTA. In order to calculate the relative hearing gain in relation to the contralateral ear, ∆PTA was divided by the initial PTA minus PTA on the contralateral side [8].

### Laboratory values

The assessment of pretreatment laboratory values included: Hematologic profile with red blood cells, hemoglobin, mean corpuscular volume (MCV), mean corpuscular hemoglobin (MCH), mean corpuscular hemoglobin concentration (MCHC), white blood cells, and platelets; glucose; electrolytes covered sodium, potassium, calcium, urea and creatinine values; the inflammation parameter C-reactive protein (CRP); lipid metabolism with cholesterol, low density lipoprotein (LDL), high density lipoprotein (HDL), LDL/HDL index, and finally the coagulation parameters Quick value, activated partial thromboplastin time (aPTT), and fibrinogen. The blood samples were taken before start of treatment to rule out an influence of the treatment on the laboratory values. The normal reference values for all laboratory parameters are given in Table 1. Most parameters are pathological if lower or higher than the reference range, but some are only pathological if lower than normal range (e.g. Quick value) or higher than the normal range (e.g. CRP).

**Table 1:** Blood values at time of diagnosis (n = 173)

**Parameter**	**Mean (SD)**	**Median**	**Min.**	**Max.**	**Normal range**	**Patients outside normal range (%)**
aPTT (sec)	30.3 (5.8)	29	20	56	26-36	39 (23)
Quick (%)	95.4 (24.7)	100.5	9	128	70-130	17 (10)
Fibrinogen (g/l)	3.1 (1)	3	1.3	8.7	1.8-3.5	43 (25)
Red-cell count (Tptl/l) women	4.6 (0.3)	4.6	3.5	5.5	4.1-5.1	9 (5)
Red-cell count (Tptl/l) men	4.9 (0.5)	4.9	3.7	7.2	4.5-5.9	19 (11)
Hemoglobin (mmol/l) women	8.5 (0.6)	8.5	7	10	7.6-9.5	8 (5)
Hemoglobin (mmol/l) men	9.1 (0.8)	9.2	6.1	10.7	8.7-10.9	21 (12)
Hematocrit women	0.41 (0.03)	0.4	0.34	0.48	0.35-0.45	5 (3)
Hematocrit men	0.43 (0.03)	0.43	0.32	0.49	0.36-0.48	4 (2)
White-cell count (/μl)	8462.4 (3102.6)	7600	1100	20900	4400-11300	28 (16)
Platelet count (Gpt/l)	250 (65.7)	245	89	610	150-360	9 (5)
MCH	1.86 (0.1)	1.9	1.2	2.12	1.74-2.05	21 (21)
MCHC	21.1 (0.6)	21.1	18.7	22.5	19.7-22.1	7 (4)
MCV	88.2 (4.8)	88	63	99	80-96	8 (5)
Glucose (mmol/l)	6.7 (2.3)	5.9	3.6	17.4	3.9-5.8	91 (53)
Sodium (mmol/l)	140.8 (2.7)	141	130	149	135-145	10 (6)
Potassium (mmol/l)	3.9 (0.4)	3.9	2.6	5.3	3.3-4.5	23 (13)
Calcium (mmol/l)	2.4 (0.1)	2.4	2	2.9	2.2-2.6	10 (6)
Urea (mmol/l)	6.1 (2.1)	5.8	2.2	17.4	2.6-7.5	32 (19)
Creatinine (lmol/l) women	80 (17.4)	78	50	189	58-96	6 (4)
Creatinine (lmol/l) men	96.3 (21.3)	92	57	182	72-127	14 (8)
C-reactive protein (mg/l)	4.7 (15.3)	1.9	1.9	188.2	≦ 7.5	12 (7)
Cholesterol (mmol/l)	5.6 (1.1)	5.6	3.1	9.3	≦ 5.2	65 (38)
LDL (mmol/l)	3.3 (1)	3.3	1.2	6.1	≦ 4.1	22 (13)
HDL (mmol/l)	1.4 (0.4)	1.3	0.8	2.8	≧ 1.0	8 (5)
LDL/HDL	2.5 (0.9)	2.4	0.6	5.3	≦ 4.1	4 (2)
Triglycerides (mmol/l)	1.81 (1.1)	1.4	0.4	7	≦ 1.7	39 (23)

SD = standard deviation; Min = Minimum; Max = Maximum; PPT = partial thromboplastin time Tpt/l = 10^3^ cells per liter; Gpt/l = 10^9^ cells per liter; MCV = mean corpuscular volume, MCH = mean corpuscular hemoglobin, MCHC = mean corpuscular hemoglobin concentration; LDL = low-density lipoprotein; HDL = high-density lipoprotein.

### Statistical analysis

If not indicated otherwise, data are presented with mean values ± standard deviation (SD). All statistical analyses were performed using IBM SPSS, version 20.0. Primary outcome criterion was absolute hearing gain (∆6PTA) dichotomized into two groups of patients (<10 dB versus ≥10 dB). The chi-square test was used to compare subgroups for ordinal parameters (e.g. gender, side, laboratory parameters dichotomized into normal versus pathologic values. The non-parametric Mann-Whitney U-test was used to compare subgroups for continuous parameters (e.g. age). Prognostic factors associated with higher frequency of hearing gain ≥10 dB with a probability value of *p* < 0.05 were included in a binary (<10 dB versus ≥10 dB hearing gain) logistic regression analysis. Nominal *p* values of two-tailed tests are reported. The significance level was set at *p* < 0.05.

## Results

### Patients’ and disease characteristics

One hundred and seventy-three (173) patients were included into the study and constituted the database for this study. Patients’ characteristics and details on SSNHL are given in Table 2. The median age was 64 years. The gender ratio was balanced (47% female and 53% male patients, respectively). There was no side predominance (53% left and 47% right ear, respectively). Four of five patients complained also of tinnitus in the affected ear. Only 14% of patients were smokers. About two of three patients (70%) had no relevant comorbidity according to Charlson comorbidity index but half of the patients showed cardiovascular risk factors.

**Table 2:** Patients’ characteristics (n = 173)

	**Number of patients (%)**
Gender	
Female	82 (47)
Male	91 (53)
Affected side	
Left	91 (53)
Right	82 (47)
First SSNHL	124 (72)
Recurrent SSNHL	49 (28)
Audiogram pattern	
Low-frequency	9 (5)
Mid-frequency	12 (7)
High-frequency	63 (36)
Flat	55 (32)
Total deafness	34 (20)
Contralateral ear	
Normal hearing	97 (56)
Abnormal hearing	40 (44)
Tinnitus, additionally	
Yes	139 (80)
No	34 (20)
Vertigo	
Yes	33 (19)
No	140 (81)
Smoking	
Yes	24 (14)
No	149 (86)
Charlson Comorbidity Index	
Index = 0	121 (70)
Index = 1	27 (16)
Index = 2	19 (11)
Index ≥ 3	6 (4)
Vascular risk profile	
Yes	93 (54)
No	80 (46)
Metabolic syndrome	
Yes	4 (2)
No	169 (98)
Final absolute hearing gain (∆6PTA)	
0 dB	24 (14)
1-19 dB	110 (64)
≥20 dB	39 (23)
	**Median, range**
Age (years)	64, 18-88
Interval onset to therapy (days)	3.5, 0-28
Hearing loss, initial (6PTA; dB)	42.5, 14.2-110
Hearing loss, final (6 PTA; dB)	30, 0.8-105.8
Hearing gain, absolute (∆6PTA; dB)	9, 0-100
Hearing loss, contralateral ear, initial (6PTA)	17.5, 0-120

Due to the 6PTA, the initial hearing loss was 50.6 ± 27.2 dB. The contralateral ear had a 6PTA of 21.2 ± 15.7 dB. The contralateral ear had a 6PTA of <20 dB, i.e. a normal hearing result, in 56% of the cases. Three patients were deaf on the contralateral ear. The interval between onset of hearing loss and begin of in-patient therapy was 5.7 ± 6.1 days. The average follow-up period, i.e. the time from first to last audiogram without further hearing improvement, was 51.0 ± 44.9 days (range: 10 – 280 days).

### Overall recovery

The absolute hearing gain between the initial audiogram and the final audiogram was 15.6 ± 20.1 dB. The mean relative hearing gain was 27.6 ± 23.7%. The mean relative hearing gain in relation to the contralateral side was 49.4 ± 45.6%. Eighty-one patients (47%) had a final hearing gain of ≥10 dB. Twenty-nine patients (17%) had a relative hearing gain of ≥50%. Seventy-two patients (42%) had a relative hearing gain in relation to the contralateral side of ≥50%.

### Prognostic impact of clinical and laboratory parameter

An overview about the serology results at time of diagnosis is presented in Table 1. Blood parameters were very variable in the study sample. About half of the patients had elevated glucose values. About one third had elevated cholesterol and a about quarter elevated triglyceride values. One quarter showed a hyperfibrinogenemia.

The univariate analysis on prognostic factors for better outcome is summarized in Table 3. The following clinical parameters were significantly correlated to better outcome (≥10 dB absolute hearing gain): Low-frequency hearing loss had a better outcome than other audiogram patterns (*p* <0.0001). Start of inpatient treatment <4 days after onset was better than a delayed treatment ≥4 days after onset (*p* = 0.018). First SSNHL had a better outcome than recurrent SSNHL (*p* = 0.001), and initial hearing loss ≥ 60 dB had a better outcome than an initial loss < 60 dB (*p* < 0.0001). Two laboratory parameters had influence on the outcome: a quick value lower than the reference values (*p* = 0.040); and a hyperfibrinogenemia (*p* = 0.007).

**Table 3:** Prognostic influence of clinical and serologic parameters on hearing gain (∆6PTA) ≥10 dB absolute hearing gain

**Parameter**	**Δ6PTA <>10 dB** ***p***
Gender	0.160
Age	0.176
Side	0.624
Tinnitus	0.133
Vertigo	0.574
Smoker	0.586
Comorbidity (Charlson Index ≥1)	0.435
Vascular risk factor	0.402
Low-frequency hearing loss	**<0.0001**
Start of inpatient treatment <4 days after onset	**0.018**
Interval between first and last audiogram	0.065
First SSNHL	**0.001**
Contralateral ear with normal hearing	0.159
Initial hearing loss ≥ 60 dB	**<0.0001**
aPTT, normal	0.905
Quick, lower than normal	**0.040**
Fibrinogen (g/l), high (hyperfibrinogenemia)	**0.007**
Red-cell count (Tptl/l) women	0.611
Red-cell count (Tptl/l) men	0.593
Hemoglobin (mmol/l) women	0.901
Hemoglobin (mmol/l) men	0.946
Hematocrit women	0.727
Hematocrit men	0.086
White-cell count (/μl)	0.433
Platelet count (Gpt/l)	0.749
MCH	0.456
MCHC	0.445
MCV	0.582
Glucose (mmol/l)	0.078
Sodium (mmol/l)	0.656
Potassium (mmol/l)	0.164
Calcium (mmol/l)	0.273
Urea (mmol/l)	0.694
Creatinine (lmol/l) women	0.116
Creatinine (lmol/l) men	0.497
C-reactive protein (mg/l)	0.380
Cholesterol (mmol/l)	0.088
LDL (mmol/l)	0.131
HDL (mmol/l)	0.922
LDL/HDL	0.367
Triglycerides (mmol/l)	0.970

p values in bold are p values below 0.05, i.e. significant p values.

Multivariate analysis revealed that first SSNHL (*p* = 0.004), start of inpatient treatment <4 days after onset (*p* = 0.015), initial hearing loss ≥ 60 dB (*p* = 0.001), and hyperfibrinogenemia (*p* = 0.032) were independent prognostic factors for better hearing gain (Table 4).

**Table 4:** Multivariate binary logistic regression analysis on independent prognostic factors for better outcome measured as absolute hearing gain ≥ 10 dB

**Parameter**	**B**	**S.E.**	**Wald**	***p***	**Odds ratio**	**95% CI lower**	**95% CI upper**
First SSNHL	-1.280	0.445	8.291	**0.004**	3.597	1.506	8.621
Low-frequency type	1.238	0.692	3.205	0.073	3.450	0.889	13.389
Quick lower than reference value	0.388	0.669	0.337	0.562	1.474	0.397	5.469
Fibrinogen high (hyperfibrinogenemia)	-0.967	0.451	4.595	**0.032**	2.631	1.086	6,369
Interval between onset and therapy begin <4 days	0.912	0.375	5.915	**0.015**	2.489	1.194	5.191
Initial hearing loss ≥60 dB	-1.482	0.463	10.243	**0.001**	4.406	1.776	10.869

p values in bold are p values below 0.05, i.e. significant p values.

## Discussion

We analyzed 173 patients with unilateral SSNHL treated within four years with a standardized treatment protocol. Interested in predictors of the prognosis, we focused not only on clinical and audiological data like in several previous studies, but included also all laboratory values of clinical routine into the univariate and multivariate analysis. Interestingly, two serologic markers with influence on the rheology of the blood, a lower quick value (<70%) and a hyperfibrinogenemia (fibrinogen > 3 g/l), were associated with better outcome.

In comparison to other studies, the observed median initial hearing loss was high with 42.5 dB. Absolute median hearing gain after combined prednisolone plus pentoxiphylline therapy was low with 9 dB. The relative hearing was 49%. Including also only SSNHL of ≥30 dB and using carbogen inhalation and prednisone orally, Cvorovic et al. recently reported for 541 patients a 15.1 dB absolute hearing gain and a relative hearing gain of 47%, i.e. in the range of the present study [5]. Using a comparable treatment regime in one study arm, a recent prospective trial reported an equivalent relative hearing gain of 43% [9]. A spontaneous hearing recovery rate without treatment for SSNHL of more than 25 dB and a relative hearing gain of 47-63% is reported [10-12]. We hypothesize that a negative selection bias is responsible for high initial hearing loss and the relative less pronounced hearing gain in the present study. First, only patients with sudden hearing loss of ≥30 dB were included. Second, inpatient treatment is mainly intended (and only covered by health insurance) in Germany if outpatient treatment fails to improve hearing within the first days after onset of SSNHL or if other symptoms like vertigo or severe hearing impairment on the contralateral side are existent. In the present study sample half of the patients had unsuccessful outpatient treatment before admission for inpatient treatment.

The two clinical factors: start of inpatient treatment <4 days after onset and first SSNHL were associated with better outcome. Furthermore, two audiological factors: low-frequency hearing loss and initial hearing loss ≥60 dB were related to better outcome. These results are partly in accordance to previous studies. It has been shown that hearing recovery is greatest when corticosteroid treatment is started within the first 1-2 weeks after onset of SSNHL [1,2,5,10]. Many studies revealed that low-frequency losses do better than high-frequency losses [5,10,13,14]. More severe initial hearing loss has higher probability of improvement in some studies but in other studies a lower probability [2,5,15]. In contrast to others, in present study vertigo or impaired hearing on the contralateral ear had no negative prognostic influence [2,5,3]. The reason why vertigo had no influence in the present study might be that patients with acute vestibular deficits elicited by caloric testing were strictly excluded.

If at all of interest, laboratory investigations were mainly analyzed on their role as risk factors for SSNHL. For instance, hypercholesterolemia and hyperglycemia were observed more frequently in SSNHL patients than in control populations [16,17]. Consistent to that, we found a hypercholesterolemia in 38% and a hyperglycemia in 53% of the patients at the time of diagnosis (cf. Table 1). Only a few studies have analyzed the prognostic role of laboratory values on treatment outcome of SSNHL. In two older studies, in times when CRP was not yet part of routine blood examinations, an elevated erythrocyte sedimentation rate was correlated to better outcome [2,10]. In the present study CRP values had no influence on outcome. As CRP is accepted to be more sensitive and specific for acute inflammatory reactions [18], and observing increased CRP values only for 7% of the study sample, we state that acute inflammatory reaction or an underlying inflammatory disease, respectively, is not related to at least most cases of SSNHL and therefore does not play a prognostic role.

Univariate statistical analysis exposed that a decreased Quick test value (<70%) at time of diagnosis was related to better hearing gain. The Quick prothrombin time test still is the basis for monitoring anticoagulant therapy in many countries worldwide [19]. Unfortunately, International normalized ratio (INR) values were not available for the majority of patients. INR values would have the advantage that the data would have been directly comparable to data from other laboratories. Seventeen (10%) of the patients with SSNHL (initial hearing loss of these patients was 65.4 ± 30.1 dB; 7 patients with initial loss ≥60 dB) had a decreased Quick value because of anticoagulant therapy in accordance to anticoagulation guidelines for cardiac diseases, history of stroke, peripheral arterial disease, or venous thromboembolism. Only patients under anticoagulant therapy showed decreased Quick values. The anticoagulant therapy was sustained during treatment of SSNHL holding Quick values in the therapeutic range (data not shown). The antithrombotic effects of the anticoagulants decrease the viscosity of the plasma. We speculate that the combination of the SSNHL therapy with the anticoagulant therapy significantly improved the microcirculatory blood flow of the inner ear. In turn, this supports the theory that a vascular impairment with disturbance of the inner ear microcirculation is at least in some patients with SSNHL a causative factor [20,21].

Even more striking was the prognostic effect of fibrinogen at time of diagnosis on the hearing gain as this parameter remained also significantly relevant in the multivariate analysis. A quarter of patients had elevated fibrinogen values at time of SSNHL diagnosis. It has been widely accepted that hyperfibrinogenemia is an independent risk factor for cardiovascular diseases [2]. Fibrinogen is the substrate for thrombin and represents the final step in the coagulation cascade and is essential for platelet aggregation [22]. Furthermore, hyperfibrinogenemia seems to be a risk factor for SSNHL [23,24]. This was the basis to introduce fibrinogen apheresis as treatment option for SSNHL [9]. Recently, it has been shown in guinea pigs that acute hyperfibrinogenemia has a direct negative impact on the cochlear microcirculation [25]. We hypothesize that patients with hyperfibrinogenemia in the present study sample had a better outcome because a vascular factor/event triggered the SSNHL. The treatment regime used primarily was designed to improve the rheological blood performance with the aim to improve the cochlear microcirculation [26].

If fibrinogen is qualified to be a biomarker for treatment selection has to be proven by further prospective trials. In deployment of the assumption that SSNHL is an umbrella term for a disease with several causative factors, such biomarkers are needed at least to select patients with vascular origin of SSNHL as only these patients can profit optimally from vascular therapy regimes.

## Conclusion

The presented cohort study on 173 patients with SSNHL revealed that beside clinical and audiological factors also the laboratory markers: decreased Quick test value and a hyperfibrinogenemia were positive prognostic markers for better outcome using a treatment regime mainly intending to improve the cochlear microcirculation. Therefore, hyperfibrinogenemia is not only a risk factor for SSNHL but also a positive prognostic marker of outcome when using a rheological regime to treat SSNHL. Especially fibrinogen seems to be an interesting candidate as biomarker for better patient selection for treatment regimens of SSNHL focusing on the refinement of cochlear microcirculation.

## Abbreviations

SSNHL: Sudden sensorineural hearing loss; PTA: Pure tone audiometry; MCV: Mean corpuscular volume; MCH: Mean corpuscular hemoglobin; MCHC: Mean corpuscular hemoglobin concentration; CRP: C-reactive protein; LDL: Low density lipoprotein; HDL: High density lipoprotein; aPTT: Activated partial thromboplastin time; SD: Standard deviation; Min: Minimum; Max: Maximum; PPT: Partial thromboplastin time; Tpt/l: 10^3^ cells per liter; Gpt/l: 10^9^ cells per liter; BERA: Brainstem electrical response audiometry; MRI: Magnetic resonance imaging.

## Competing interests

There is no competing interest. The authors confirm that they do not have any financial relationship concerning this research.

The authors indicate that they have no a financial relationship with any organization or company mentioned in the manuscript. The research was not sponsored by a third party.

## Authors’ contribution

OGL and CW had the idea for the study. OGL and MK drafted the manuscript. JW performed the data collection. OGL and JW performed the statistical analysis. OGL designed tables and figures. All authors read and approved the final manuscript.

## Pre-publication history

The pre-publication history for this paper can be accessed here:

http://www.biomedcentral.com/1472-6815/14/6/prepub
